# Supplementary material for: Genome organization by SATB1 binding to base-unpairing regions (BURs) provides a scaffold for SATB1-regulated gene expression
Source: eLife. 2025 Oct 2;14:RP105915. doi: 10.7554/eLife.105915 (PMC12490858; doi:10.7554/eLife.105915)
Supplement: Figure 3—source data 1. [file elife-105915-fig3-data1.pdf]

Figure 3-source data 1

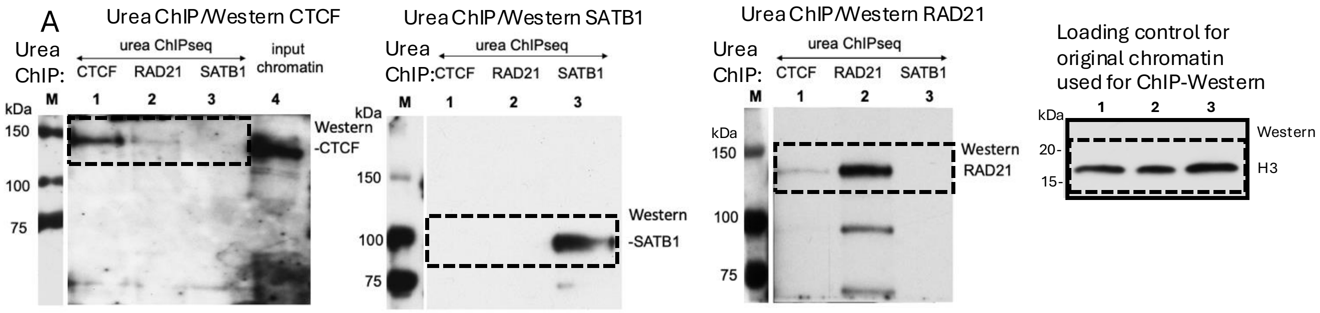

Figure 3 Source Data 1. Original membranes corresponding to Figure 3, panel C. The areas marked by dotted brackets are used for Figure 3C. Marker lanes (M) are either from the same gel or from another gel that was run in parallel in the same vertical dual gel electrophoresis chamber. Input chromatin in the leftmost panel indicates whole urea-purified chromatin before immunoprecipitation.
