# Supplementary figures and images for: Genome organization by SATB1 binding to base-unpairing regions (BURs) provides a scaffold for SATB1-regulated gene expression
Source: eLife. 2025 Oct 2;14:RP105915. doi: 10.7554/eLife.105915 (PMC12490858; doi:10.7554/eLife.105915)

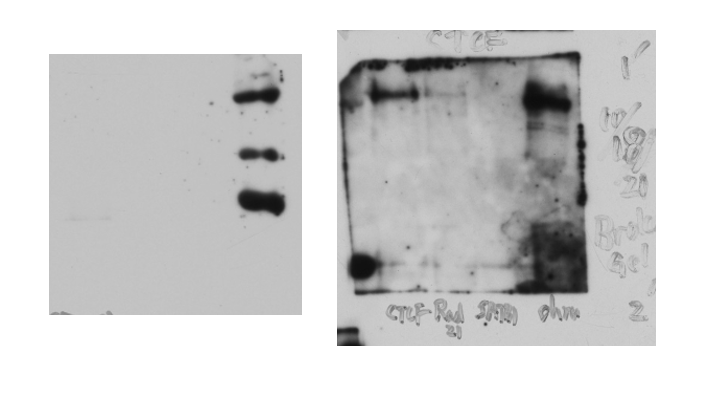

Supplement: Figure 3—source data 2. [file elife-105915-fig3-data2.zip › urea ChIP-Westen CTCF.tiff]

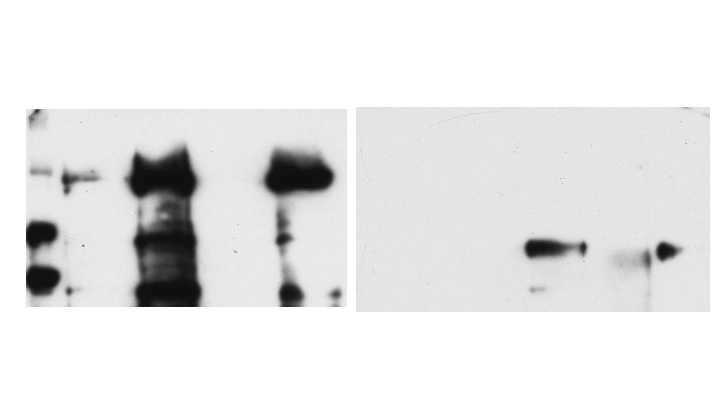

Supplement: Figure 3—source data 2. [file elife-105915-fig3-data2.zip › urea ChIP-Western SATB1.tiff]

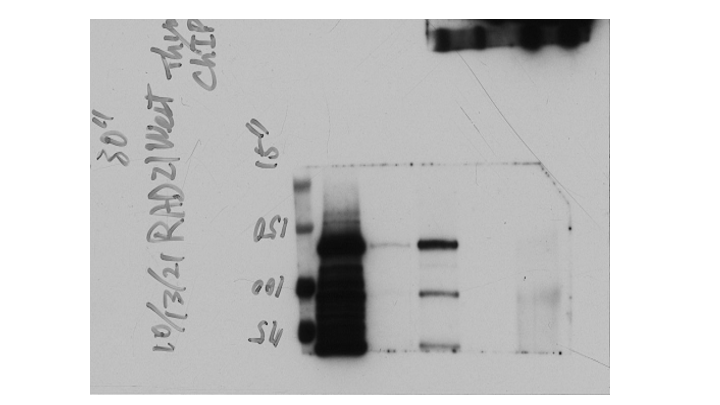

Supplement: Figure 3—source data 2. [file elife-105915-fig3-data2.zip › urea ChIP-Western RAD21.tiff]

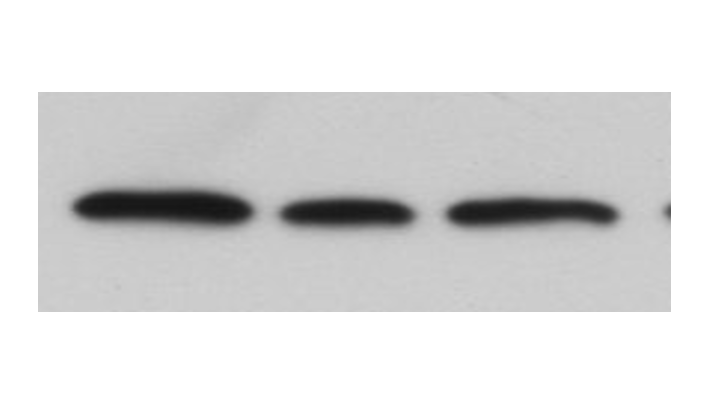

Supplement: Figure 3—source data 2. [file elife-105915-fig3-data2.zip › H3 loading control.tiff]
